# Effects of Particle Size and Extraction Methods on Cocoa Bean Shell Functional Beverage

**DOI:** 10.3390/nu11040867

**Published:** 2019-04-17

**Authors:** Olga Rojo-Poveda, Letricia Barbosa-Pereira, Lívia Mateus-Reguengo, Marta Bertolino, Caroline Stévigny, Giuseppe Zeppa

**Affiliations:** 1 Department of Agriculture, Forestry and Food Sciences (DISAFA), University of Turin, 10095 Grugliasco, Italy; olgapaloma.rojopoveda@unito.it (O.R.-P.); liviareguengo@gmail.com (L.M.-R.); marta.bertolino@unito.it (M.B.); 2 RD3 Department-Unit of Pharmacognosy, Bioanalysis and Drug Discovery, Faculty of Pharmacy, Université Libre de Bruxelles, 1050 Brussels, Belgium; Caroline.Stevigny@ulb.be; 3 Department of Analytical Chemistry, Nutrition and Food Science, Faculty of Pharmacy, University of Santiago de Compostela, 15782 Santiago de Compostela, Spain

**Keywords:** cocoa by-product, functional food, polyphenols, α-glucosidase inhibition, antidiabetic capacity, antioxidant capacity, methylxanthines

## Abstract

One of the main by-products in cocoa industry is the cocoa bean shell (CBS), which represents approximately 12–20% of the bean. This product has been suggested as a food ingredient because of its aroma and high dietary fiber and polyphenol contents. The purpose of this work was to evaluate the effects of the CBS particle size and extraction methods on the chemical composition and consumer acceptance of a functional beverage, in order to find the best combination of technological parameters and health benefits. Five particle sizes of CBS powder and six home techniques were used for beverage preparation. The influence of these factors on the physico-chemical characteristics, methylxanthine and polyphenolic contents, antioxidant and antidiabetic properties, and consumer acceptance was evaluated. Total phenolic content values up to 1803.83 mg GAE/L were obtained for the beverages. Phenolic compounds and methylxanthines were identified and quantified by HPLC-PDA. These compounds may be related to the high antioxidant capacity (up to 7.29 mmol TE/L) and antidiabetic properties (up to 52.0% of α-glucosidase inhibition) observed. Furthermore, the consumer acceptance results indicated that CBS may represent an interesting ingredient for new functional beverages with potential health benefits, reducing the environmental and economic impact of by-product disposal.

## 1. Introduction

According to the International Cocoa Organization (ICCO), each year, more than 4000 tons of cocoa beans are processed and consumed worldwide [1]. Considering that the main products of cocoa are obtained from its roasted bean, which represents only 10% of the total weight of the fruit, cocoa processing produces a large amount of vegetal residue. Besides being expensive, the disposal of these by-products can be harmful for the environment because they contain potentially phytotoxic polyphenols [2] and high concentrations of theobromine, which may be toxic for non-human mammals [3]. Such underutilization of residual biomass can be overcome by the development of an added-value foodstuff based on cocoa by-products, particularly the cocoa bean shell (CBS), which represents 12% to 20% of the cocoa bean [4]. CBS has been reported to be a considerable source of proteins and dietary fiber, with low fat content in comparison with cocoa beans [5] but with a similar profile of volatiles [6]. Considering that CBS is a final-stage by-product from cocoa processing, it appears to be an economical, organoleptic, and nutritionally rewarding substance for the transformation of cocoa bean industries. Most of the research into CBS utilization is related to animal feeding and, despite the presence of theobromine, CBS has positive effects on fortified diets for ruminants, pigs, and poultry [3], among other animals. Besides, CBS has been applied as an additive in organic fertilizer [7], as biomass for biogas production [8], or as a pectin source [5], among other applications. The application of this cocoa by-product to food has attracted some attention due to its nutritional characteristics and high concentration of phenolic compounds, mainly flavonoids [9]. In recent years, some studies in the food research field were published, suggesting CBS as a food ingredient [4]. This research interest can be linked to the sustainability and bioeconomy framework of the modern food and agricultural industries, leading to the valorization of functional foods developed from by-products, as an opportunity to make healthier foods. Functional foods are similar to conventional ones in that they are part of a standard diet and consumed on a regular basis and in regular amounts. A functional food is claimed to have proven benefits for the maintenance or promotion of a state of well-being or health or a reduction in the risk of a pathological process or disease [10]. Although this market niche is not well defined, by influencing the data on global sales, functional foods irrefutably represent a top trend in the food industry.

Because of their antioxidant capacity, phenolic compounds may be capable of protecting cell components from oxidative damage, thus limiting the risk of several diseases associated with oxidative stress, for instance, diabetes [11]. The relevance of polyphenols to the management of blood glucose is mainly due to their inhibition of digestive enzymes involved in the metabolism of carbohydrates (α-glucosidase and α-amylase) [9]. Even if these phenolic compounds are poorly absorbed, they can still act on membrane-bound enzymes located in the intestinal epithelium. Moreover, other mechanisms of action of polyphenols on glucose uptake, after ingestion of carbohydrate-rich meals, are being studied [12]. The presence of bioactive components in CBS and its sustainable character may arouse interest in such a product as a potential ingredient in the functional-beverage industry. Despite their resource-rich matrix, the effective extraction of plant bioactive compounds and their concentrations in the final product can be influenced by processing or preparation methods.

Unground CBS has already been utilized as one of the ingredients for commercialized herbal infusion bags, such as the “ChocoTea” infusion bags from Valberbe^®^ or the “Choco” tisane bags from YogiTea^®^. Nonetheless, other preparations destined for the use of this by-product in home-based beverage-making techniques have not been proposed yet. In this paper, several CBS preparations were developed to be employed in six diffused techniques for coffee home-preparation available at a consumer level, such as the Moka, Neapolitan flip, American, Espresso, Capsule, and French press coffee makers. Instead of coffee powder, CBS at different grinding degrees (GDs) was employed to find the optimal GD for each extraction technique. The aim of this study was to find the best combination of the CBS GD and the beverage preparation technique in order to obtain a new functional beverage with the optimal chemical composition, biological effects (antioxidant and antidiabetic properties), and sensory characteristics.

## 2. Materials and Methods

### 2.1. Chemicals

Folin & Ciocalteu’s phenol reagent, sodium carbonate (≥99.5%), 2,2′-diphenyl-1-picrylhydrazyl (95%) (DPPH), 6-hydroxy-2,5,7,8-tetramethylchroman-2-carboxylic acid (97%; trolox), vanillin (99%), (+)-catechin hydrate (>98%), methanol (≥99.9%), hydrochloric acid (fuming 37%), aluminum chloride (99%), sodium nitrite (≥99%), α-glucosidase from intestinal acetone powders from rat, p-nitrophenyl-α-D-glucopyranoside (≥99%; p-PNG), acarbose (≥95%), potassium phosphate monobasic (≥99%), formic acid (≥98%), quercetin-3-O-glucoside (≥90%; Q-3-G), theobromine (≥98.5%), caffeine (≥98.5%), and quercetin (≥98.5%) were provided by Sigma-Aldrich (Milan, Italy). Potassium phosphate dibasic (≥ 98%) was acquired from Carlo Erba (Milan, Italy). Gallic acid, ethanol (≥99.9%), sodium hydroxide (1 M), (-)-epicatechin (>90%), procyanidin B1 (≥98.5%; PCB1), procyanidin B2 (≥98.5%; PCB2), protocatechuic acid (>97%), caffeic acid (≥95%), and vanillic acid (≥99%) were supplied by Fluka (Milan, Italy). Ultrapure water was prepared in a Milli-Q filter system (Millipore, Milan, Italy).

### 2.2. Samples

CBS from São Tomé cocoa beans (Forastero variety) was kindly supplied by Pastiglie Leone S.r.l. (Turin, Italy). The CBS was divided into different grain sizes using a BA200N vibrating sieve (CISA, Barcelona, Spain). Five GDs were obtained: above 4000 μm (GD1), 2000–4000 μm (GD2), 1000–2000 μm (GD3), 500–1000 μm (GD4), and 250–500 μm (GD5).

The chemical and nutritional characterization of CBS was carried out according to Bertolino et al. [13].

### 2.3. Preparation of Beverages

The beverages were prepared by six techniques available to consumers, i.e., Moka Express 6 cups (Bialetti, Brescia, Italy), Neapolitan flip coffee pot (Ilsa, Turin, Italy), American coffee maker Cucina HD 7502 (Phillips, Milan, Italy), Espresso Saeco HD 8423/11 (Phillips, Milan, Italy), Capsule LM 3100 (AEG, Milan, Italy), and French press Kaffe (Ikea, Collegno, Italy). Still mineral water (Valmora, Luserna San Giovanni, Italy) was used for the beverage production. Each of the GDs was tested with all the coffee makers, resulting in 30 beverages. Three production batches were generated for each beverage.

The water quantities employed varied for each technique following the established rules for the use of the different machines. For Espresso and Capsule techniques, the initial volumes of water are unknown because both machines operate with a continuous inlet of water and therefore it is possible to define only the final volume of the obtained beverage. In addition, the quantities of CBS powders used for the beverage preparations were adapted to each technique and GD to obtain a technologically viable formulation.

All the beverages were centrifuged on a MPW-260R centrifuge (MPW, Warsaw, Poland) at 3075 ×*g* for 10 min and then passed through a 0.45 μm cellulose acetate filter (Carlo Erba, Milan, Italy) before analyses.

### 2.4. Analytical Procedures

#### 2.4.1. Physicochemical Analysis

A pH meter MICROpH 2002 (CRISON, Carpi, Italy) served for pH measurement.

Determination of total acidity (expressed in grams of acetic acid per liter of a beverage) was performed by potentiometric titration of 5 mL of a beverage (diluted to 50 mL with distilled water) by means of 0.01N NaOH up to pH 8.2.

The dry extract of each beverage was analyzed gravimetrically for a 5 mL sample of each beverage dried in an oven at 110 °C until constant weight.

The color analysis was conducted in transmittance mode on a CM-5 spectrophotometer (Konica Minolta, Tokyo, Japan). L*, a*, and b* CIELab parameters were used to measure the color, where L* is a coefficient of lightness ranging from 0 (black) to 100 (white), a* indicates the colors red-purple (when positive a*) and bluish-green (when negative a*), and b* denotes the colors yellow (when positive b*) and blue (negative b*). The ΔE parameter, which represents the difference between two colors [14] and its perceptibility by the human eye when ΔE > 2.5, was calculated according to the equation

$$\Delta E=\;\sqrt{{\left(L{*}_{a}-L{*}_{b}\right)}^{2}+{\left(a{*}_{a}-a{*}_{b}\right)}^{2}+{\left(b{*}_{a}-b{*}_{b}\right)}^{2}}$$

#### 2.4.2. Total Phenolic, Tannin, and Flavonoid Contents

The total phenolic (TPC), total flavonoid (TFC), and total tannin (TTC) contents were determined according to the methods described by Barbosa-Pereira et al. [15], in 96-well microplates, using a BioTek Synergy HT spectrophotometric multi-detection microplate reader (BioTek Instruments, Milan, Italy). All the measurements were performed in triplicate. For the TPC analysis, a calibration curve of gallic acid (20–100 mg/L) was constructed to quantify the concentration, which was expressed in milligrams of gallic acid equivalents per liter of a beverage (mg GAE/L). The quantification of both TFC and TTC was performed based on a standard curve of catechin (5–500 mg/L), and the concentrations were expressed in milligrams of catechin equivalents per liter of a beverage (mg CE/L).

#### 2.4.3. Antioxidant Capacity

The antioxidant capacity of the beverages was assessed by the 2,2′-diphenyl-1-picrylhydrazyl (DPPH•) radical–scavenging method described by Barbosa-Pereira et al. [15]. All the assays were conducted in triplicate in 96-well microplates with the BioTek Synergy HT spectrophotometric multi-detection microplate reader (BioTek Instruments). Antioxidant capacity was calculated as the inhibition percentage (IP) of the DPPH radical as

$$IP\;\left(\%\right)=\;\frac{\left(A_{0}-A_{30}\right)}{A_{0}}\;\times 100\;$$

where A_0_ is absorbance at the initial time point, and A_30_ is the absorbance after 30 min.

A standard curve of trolox was constructed (12.5–300 μM) for assessment of the radical-scavenging activity values, which were expressed as millimoles of trolox equivalents per liter of a beverage (mmol TE/L).

#### 2.4.4. Antidiabetic Capacity

The antidiabetic effects of the beverages were determined by the α-glucosidase colorimetric assay adapted from the method described by Kwon et al. [16].

An aliquot (50 μL) of the sample was mixed with 100 μL of α-glucosidase (10 mg/mL), prepared in 0.1 M phosphate buffer pH 6.9, and incubated for 5 min. After that, 50 μL of substrate p-PNG at 4 mM (prepared in the phosphate buffer) was added, and the solution was mixed. The solution was incubated for 30 min at 37 °C, and then absorbance was measured at 405 nm against a blank control. Acarbose at 0.5 mM (half-maximal inhibitory concentration, IC_50_) served as a positive control, and the antidiabetic capacity was expressed as the α-glucosidase inhibition percentage. All of the measurements were conducted in triplicate in 96-well microplates, using the BioTek Synergy HT spectrophotometric multi-detection microplate reader (BioTek Instruments).

#### 2.4.5. RP-HPLC-PDA Analysis

Characterization of the polyphenols contained in the beverages was performed by means of reversed-phase high-pressure liquid chromatography with a photodiode array detector (RP-HPLC-PDA) Thermo-Finnigan Spectra System (Thermo-Finnigan, Waltham, MA, USA). The instrument was equipped with a P2000 binary gradient pump, SCM 1000 degasser, AS 3000 automatic injector, and Finnigan Surveyor PDA Plus detector. Instrument control, data collection, and data processing were performed using the ChromQuest software, version 5.0 (Thermo-Finnigan, Waltham, MA, USA).

For separation of compounds, a reverse-phase Kinetex Phenyl-Hexyl C18 column (150 × 4.6 mm internal diameter and 5 μm particle size; Phenomenex, Castel Maggiore, Italy) was utilized at 35 °C.

Two solvents served as a mobile phase: water containing formic acid at 0.1% *v*/*v* (solvent A) and 100% methanol (solvent B). The sample injection volume was 10 μL. To separate the different compounds, gradient elution at a flow rate of 1 mL/min was conducted during 45 min as follows: minutes 0–2, 90% A and 10% B; minutes 2–18, a linear gradient from 10% to 50% B; minutes 18–40, a linear gradient from 50% to 80% B; minutes 40–42, a linear gradient from 80% to 90% B; and minutes 42–45, a linear gradient until 90% A and 10% B were reached.

Detection was carried out via continuous scanning of wavelengths between 200 and 400 nm. Methylxanthines (theobromine and caffeine) were quantified at 272 nm, protocatechuic acid at 293 nm, caffeic acid at 325 nm, flavan-3-ols (catechin, epicatechin, and catechin-3-O-glucoside), and procyanidins B (type B procyanidin and procyanidin B2) were quantified at 280 nm, and flavonols (quercetin, quercetin-3-O-glucoside, and quercetin-3-O-rhamnoside) at 365 nm.

The quantification was performed based on external linear calibration curves analyzed under the same conditions and the following correlation coefficients were obtained: R^2^ = 0.9995 for theobromine, R^2^ = 0.9996 for caffeine, R^2^ = 0.9999 for catechin, R^2^ = 0.9998 for epicatechin, R^2^ = 0.9997 for protocatechuic acid, R^2^ = 0.9999 for caffeic acid, R^2^ = 0.9998 for procyanidin B1 (PB1), R^2^ = 0.9999 for procyanidin B2 (PB2), R^2^ = 0.9996 for quercetin-3-O-glucoside (Q-3-G), and R^2^ = 0.9988 for quercetin. For catechin-3-O-glucoside, type B procyanidin, and quercetin-3-O-rhamnoside, concentrations were expressed as catechin, procyanidin B1, and quercetin-3-O-glucoside equivalents, respectively.

### 2.5. Consumer Acceptance Evaluation

For each beverage, a consumer test was carried out with 20 tasters where appearance, odor, taste, flavor, texture, overall liking, and purchase predisposition were evaluated on a nine-point hedonic scale (1 = extremely dislike, 9 = extremely like) [17]. The tests were performed in an air-conditioned room with white light at approximately 21°C.

### 2.6. Statistical Analysis

All the obtained results were subjected to analysis of variance (ANOVA) with Duncan’s post hoc test at 95% confidence level and to linear regression analysis in the Windows software called STATISTICA, version 13.3 (StatSoft Inc., Tulsa, OK, USA).

Values obtained by the consumer test were analyzed by the Kruskal–Wallis test (test H).

## 3. Results and Discussion

### 3.1. Cocoa Bean Shell—Chemical and Nutritional Composition

The chemical and nutritional composition of the cocoa bean shell employed in beverage preparation, expressed for 100 g of dried product, was as follows: protein: 20.9 g, fat: 2.3 g, carbohydrates: 7.85 g, dietary fiber: 55.1 g (42.3 g of insoluble fiber and 12.8 g of soluble fiber), water: 5.9 g, and ash: 7.9 g.

### 3.2. Beverage Yield

Thirty formulations were developed, resulting in 30 beverages with different yields, mostly depending on the CBS GD (Table 1). Yields ranged from 72.0% to 93.3% when GDs above 500 μm were used, and a notable substantial decrease in the recovery percentage was observed with a reduction in the GD, thereby leading to such values as 29.6% for the beverage prepared with the Moka and GD5. This decrease could be mostly due to the water-holding capacity of the insoluble fiber present in the CBS; this fiber became more available when the surface-to-volume ratio of the CBS powders increased. In some cases, also the larger CBS powder quantities used in order to obtain technologically realistic preparations influenced the water-holding capacity. Nevertheless, this influence may not affect the beverages obtained with other coffee makers such as the American pot or French press where the CBS quantities remain the same at all the GDs, and the reduction in the yield is due to the lower GD only.

For the lowest GD (GD5, 250–500 μm), the beverage yields decreased considerably, and this parameter, in general, lost the repeatability observed for larger GDs, mostly owing to the technological problems during beverage preparation such as machine blockage in cases where the CBS absorbed too much water or with extremely long preparation periods. Due to the aforementioned problems, the beverages obtained by the Moka, Neapolitan, Espresso, and Capsule techniques with GD5 were assumed to be not technologically viable and it was not possible to proceed with further analyses. Only the beverages obtained with the French press and American techniques were considered for GD5.

### 3.3. Physico-Chemical Characterization

#### 3.3.1. Acidity, Dry Matter, and Color

The pH and the titratable acidity results obtained for the functional beverages are shown in Table 2. The beverages had pH and titratable acidity ranging from 4.84 to 5.19 and from 0.12 to 1.64 g of acetic acid equivalents per liter of a beverage, respectively. In general, lower pH and higher acidity were observed in beverages produced with a lower GD, except for the beverages produced with the Moka and the Neapolitan techniques, which showed nonsignificant differences in pH when the GD was varied. Nevertheless, they still showed the same tendency for acidity, which increased with a decrease in particle size, probably owing to a major acid extraction when the CBS surface-to-volume ratio was increased. This tendency was not observed for the beverage produced with the French press technique where pH and acidity were found to be independent of the CBS particle size.

For all the beverages obtained by percolation techniques (Moka, Neapolitan, American, Espresso, and Capsule), the quantity of dry matter increased when the GD was reduced (Table 2) because the extraction seemed to be more effective at low GD. For French press, the quantity of dry matter did not correlate with the GD of CBS, suggesting that the extraction by this maceration technique was barely affected by the particle size of the CBS powder.

Regarding the chromatic parameters (Table 2), generally, the brightness parameter decreased with the decreasing GD due to the increase in the surface-to-volume ratio of the CBS powder, thereby allowing for better extraction of color pigments. The beverages having the lowest values of L* and therefore, darker beverages, were those obtained at the GD4 with Moka and Neapolitan techniques. Generally, both parameters a* and b* rose with the decreasing GD, as the beverages became browner. Always following a similar trend, various beverages showed significant differences when the GD was changed except for the beverages produced with the French press. This fact can be numerically explained by the ΔE parameter (data not shown), which determines whether two colors can be distinguished by the human eye (ΔE > 2.5). Considering the different CBS GDs within each technique, the beverages showed values of ΔE higher than 2.5 except for some beverages obtained with the French press technique, where ΔE_GD1-GD2_ = 2.10, ΔE_GD1-GD3_ = 1.31, and ΔE_GD2-GD5_ = 1.55, which means that these beverages had colors indistinguishable for the human eye.

#### 3.3.2. Polyphenolic Content

**Figure 1:**
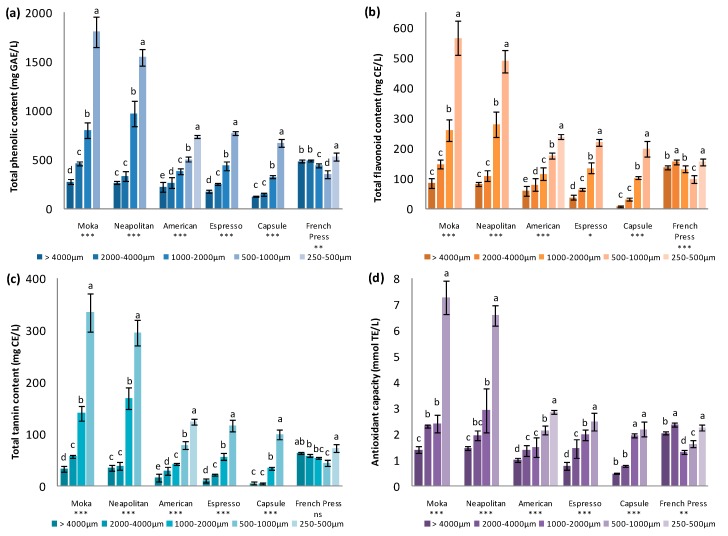
Total phenolic content (TPC), (**a**), total flavonoid content (TFC) (**b**), and total tannin content TTC (**c**), and antioxidant capacity (**d**) for the beverages produced by the six techniques at different CBS GDs; ANOVA among GDs for each technique. GAE = gallic acid equivalent, CE = catechin equivalent, and TE = trolox equivalent. Different letters indicate significant differences at *p* < 0.05. Significance: * *p* < 0.05; ** *p* < 0.01; *** *p* < 0.001; ns = not significant.

TPC, TFC, and TTC data are presented in Figure 1 (Figure 1a, Figure 1b, and Figure 1c, respectively).

TPC varied considerably among the beverages obtained by different techniques and at varied GDs, with values that ranged from 126.86 mg GAE/L for the beverage obtained by the Capsule technique at GD1 to 1803.83 mg GAE/L for the beverage obtained by the Moka technique using GD4. Except for the beverage obtained with the French press, large significant differences were detected for all the other beverages when the GD of the CBS was varied. For the beverages obtained by percolation techniques, TPC increased with a reduction in the GD, whereas for the beverage produced by the maceration technique (French press), TPC values were not influenced by the GD. The highest phenolic content was seen in beverages prepared by the Moka and Neapolitan techniques, where the results ranged from 276 mg GAE/L at GD1 to 1803.83 mg GAE/L at GD4 and from 263.67 mg GAE/L at GD1 to 1545.87 mg GAE/L at GD4, respectively. The beverage obtained by the Capsule technique manifested the lowest values of this parameter, ranging from 126.86 mg GAE/L at GD1 to 671.68 mg GAE/L at GD4. On the other hand, considering the intake, one cup (200 mL) of the beverage obtained by the French press technique at any GDs or by the American technique using CBS at GDs 3–5, provided the same quantity of polyphenols as one cup (60 mL) of the beverages obtained by the Moka or the Neapolitan techniques with CBS at GD4. The different values of TPC obtained for the beverages ranged between various values presented in previous studies, where beverages thought to have ‘high-polyphenol content’ were evaluated. The values of TPC in the present study were in most cases even higher than those found by Zujko & Witkowska [18] for drinking chocolate (600 mg GAE/L) or hot cocoa (300 mg GAE/L), but also higher in some cases than those of different tea types such as white tea (1040 mg GAE/L), green tea (850 mg GAE/L), black tea (720 mg GAE/L), and red tea (380 mg GAE/L). Reported TPC values for red wine (2410 mg GAE/L) and white wine (260 mg GAE/L) [18] were also within the range of the values obtained for the beverages studied in the present work. Regarding the values reported for some fruit juices by Gardner et al. [19] such as orange (755 mg GAE/L), apple (339 mg GAE/L), or pineapple juice (358 mg GAE/L), the levels of total phenols in CBS beverages were always between these values or even higher.

Flavonoids were the main compounds that contributed to TPC, constituting from 20.8% to 34.7% of this value, depending on the preparation technique but these contributions remained constant at different GDs within each technique. TFC and TPC were highly correlated (*r* = 0.9965), and thus the former followed the same tendencies of abundance depending on the technique and GD. In this way, the highest value of TFC was seen in the beverages obtained by the Moka technique at GD4 (566.42 mg CE/L), followed by the beverage obtained by the Neapolitan technique with GD4 (489.43 mg CE/L). Beverages obtained by the two techniques had higher values at each GD compared with the other techniques except for the French press, for which the beverages showed slight differences with the variation of the GD. The TFC values of the beverages produced by the French press technique ranged between 97.98 and 155.63 mg CE/L and therefore had the highest values when larger CBS particle sizes were chosen. Again, the lowest values were observed for the Capsule beverage, which had TFC between 8.78 and 198.47 mg CE/L, which increased with a reduction in the CBS particle size.

Values of TTC accounted for 3.4% to 19.2% of TPC with a significantly high correlation (*r* = 0.9968). Contrary to what was observed between TPC and TFC, the percentage of TTC’s contribution to TPC increased with a reduction in the GD within the values obtained for each technique, except for the beverages produced by the maceration technique, which maintained the contribution of the tannin content (12.0% to 13.9%) to TPC values independently of the GD. This fact can be noticed as the differences between big and small CBS particle sizes in TTC values become higher than those of TPC and TFC. CBS particle size could have an influence on selective extraction of some polyphenol groups for the percolation techniques, as could be the case for tannins. Tannins are normally larger molecules than flavonoids and therefore, a decrease in the particle size could facilitate their extraction compared to that of flavonoids, which were already extracted using the CBS powder with high particle sizes. In this way, greater increases were observed for TTC within the same technique when the particle size was reduced in comparison with those observed for TPC and TFC. Nevertheless, the highest concentrations of tannins were again detected in the Moka and Neapolitan beverages at GD4 (334.64 and 296.06 mg CE/L, respectively) and the lowest values of TTC were observed in the Capsule beverage, which ranged from 5.18 to 99.86 mg CE/L.

More than 30 polyphenolic compounds that may contribute to the above values were detected and quantified by HPLC analysis. Only the concentrations determined for the main cocoa marker phenolic compounds and those showing the highest concentrations are given in Table 3. Belonging to the group of phenolic acids, protocatechuic acid, and caffeic acid were found in CBS beverages, with the former being the most abundant. Both phenolic acids have already been quantified in cocoa bean and chocolate samples [20,21]. Nonetheless even if the caffeic acid was present at lower concentrations than protocatechuic acid, in the CBS beverages it showed higher levels (with respect to protocatechuic acid) than those found in chocolate 100% cocoa made from Sao Tome cocoa beans (Forastero variety) studied by Rodríguez-Carrasco et al. [21]. These results indicate that the proportions of these two components diverge in the CBS with respect to the cocoa bean. For protocatechuic acid, the concentrations depending on the technique and GD used to produce the beverages followed a trend similar to the one already observed. The highest concentration was obtained in the beverage produced by the Moka technique using the minimum GD (18.14 mg/L) while the lowest values were found for the Capsule beverage (ranging from 1.32 to 10.80 mg/L) and the beverage produced by the American technique (ranging from 2.91 to 7.23 mg/L). Similar data were obtained for the French press beverage regardless of the particle size (ranging from 5.06 to 7.70 mg/L). As for caffeic acid, the lowest concentrations were seen in the beverage produced by the American technique, with values ranging from 0.15 to 0.33 mg/L. A slight decrease in the caffeic acid concentration was observed in the beverages prepared by the French press technique in comparison with the other techniques.

As for flavan-3-ols, we detected and quantified catechin, epicatechin, and catechin-3-O-glucoside in all the beverages; these are three characteristic flavan-3-ols for cocoa beans and chocolate already reported in CBS [15]. They can be found as free monomers or forming condensed tannins as monomeric constituents [22]. Variable concentrations were noted for these compounds, with catechin-3-O-glucoside being the most abundant, followed by epicatechin and catechin. Nevertheless, the concentrations obtained for both catechin and epicatechin were lower than those obtained with other types of extraction procedures where organic solvents were used as in the work of Hérnandez-Hérnandez et al. [23]. Catechin and epicatechin showed low solubility in water, even when pressure and high temperatures were applied in the present work, whereas catechin-3-O-glucoside was present at higher concentrations, probably because of the water solubility ensured by the glycoside group. As a flavonoid compound, epicatechin significantly correlated with the results obtained for both TPC and TFC (*r* = 0.9628 and *r* = 0.9574, respectively).

Two procyanidins of type B were detected and quantified by HPLC analysis. Procyanidins are flavan-3,4-diols, generally forming condensation compounds with epicatechin at 4–8 or 4–6 bonds [22]. Type B procyanidin was found to be the polyphenolic compound at the highest concentration among those quantified in the studied samples. This compound was present at the highest concentrations in both Moka and Neapolitan beverages with the smallest CBS particle size (36.30 and 27.10 mg procyanidin B1 eq/L, respectively) and with the lowest values for the beverages produced by the Capsule and American techniques; the concentration increased with a decrease in the GD. As epicatechin, this compound highly correlated with both TPC and TFC (*r* = 0.9621 and *r* = 0.9572, respectively). Procyanidin B2 was also found at its highest concentrations in the Moka and Neapolitan beverages, and at lower concentrations in the Capsule and American ones. These results followed the trend of an increasing concentration when the GD was decreased for almost all the beverages obtained by the percolation techniques; and an intermediate constant concentration was observed for the maceration technique, independently of the GD.

Finally, three flavonols were quantified in the CBS beverages, quercetin and two of its glycoside derivates: quercetin-3-O-glucoside and quercetin-3-O-rhamnoside. Quercetin and its derivates are part of the more abundant and recurrent flavonoids in foods known for their bitter flavor [22].

All these polyphenolic compounds, which possess antioxidant properties, could be beneficial in terms of prevention of diseases related to oxidative stress. Therefore, it is highly important for humans to consume them with nutrition. The new beverages (based on CBS) developed in this study may be an interesting source of these bioactive compounds with potential health benefits. 

#### 3.3.3. Methylxanthines

The concentrations of theobromine and caffeine evaluated by HPLC are given in Table 3. The amounts of theobromine were approximately 5–7-fold higher than those of caffeine. The concentrations of both methylxanthines significantly increased with a reduction in the GD for all the beverages obtained by percolation techniques. In general, the beverages that manifested the highest concentrations were those obtained by the Moka and Neapolitan methods, ranging from 147.33 to 703.79 mg theobromine/L and 25.44 to 124.84 mg caffeine/L for the former, and from 127.84 to 583.12 mg theobromine/L and 19.17 to 102.98 mg caffeine/L for the latter. The observed caffeine contents were lower than those observed for other kinds of beverages such as coffee (567 mg/L), mate (520 mg/L) [24], matcha (300 mg/L), loose leaf teas (99.21–296.86 mg/L), or bagged teas (151.73–246.71 mg/L) [25]. Nonetheless, the theobromine contents of CBS beverages are significantly higher than those observed for the same beverages, showing such amounts as 12.18 mg theobromine/L for matcha, 7.55–86.18 mg/L for loose leaf teas, and 21.58–66.91 mg/L for bagged teas [25].

Nevertheless, considering the expected intake of each type of beverage (60 mL for Moka, Neapolitan, Espresso, and Capsule and 200 mL for French press and American), the largest amounts of theobromine and caffeine would be consumed with the beverage produced by the French press technique, for which up to 60 mg of theobromine and 9 mg of caffeine would be consumed with each expected dose.

Cocoa is known for stimulating the brain due to the presence of theobromine and caffeine. As mentioned above, these two methylxanthines are also present in the CBS, the former being notably more abundant than the latter. They both influence alertness and mood in a positive way, acting on the central nervous system, and thus, may partly account for cocoa acceptance by consumers. Besides, several beneficial biological activities are linked to these methylxanthines such as the anticarcinogenic, antiobesity, antioxidant, antitumor, diuretic, or energizer effects of caffeine or the cAMP-inhibitory (with IC_50_ = 0.06 mg/mL), cAMP-phosphodiesterase-inhibitory, diuretic (when consumed at 300–600 mg/day), stimulant, or myorelaxant activities of theobromine [24]. Theobromine is also linked to the beneficial effects of cocoa consumption because of various other benefits associated with it, all of them without some of the unwanted effects of caffeine [26]. Usmani et al. [27] observed that a single 1000 mg dose of theobromine has a significantly greater antitussive effect on humans than a single 60 mg dose of codeine, an opioid drug whose clinical use is limited due to its unacceptable side effects, which could be avoided with an alternative theobromine treatment. Neufingerl et al. [28] reported that 850 mg of theobromine per day increases serum high-density lipoprotein cholesterol concentrations by 0.16 mmol/L. Another example of theobromine consumption benefits has been demonstrated by Kargul et al. [29], who showed that application of theobromine at concentrations of 100 and 200 mg/L to teeth could significantly protect enamel surface via a cariostatic effect, thus being an alternative to fluoride treatments. According to these data, CBS is likely to represent the proper combination of both methylxanthines in order to exert all the aforementioned beneficial actions without the secondary effects of big doses of caffeine, such as tachycardia or increased blood pressure if consumed at doses over 250 mg [30]. Regarding the above-mentioned examples of theobromine’s benefits, one single expected dose of the CBS beverage would not yield the needed levels to observe the effects on high-density lipoprotein cholesterol level or the antitussive properties but it would clearly contribute to these benefits. Moreover, further optimizations or extract concentrations of the prepared beverages could be proposed to improve their functionality. Additionally, harmful levels of caffeine will not be an issue when expected doses of the beverages are consumed.

### 3.4. Biofunctional Characteristics

Polyphenols, as antioxidants, chelators of divalent cations, or inhibitors of enzymatic activities, have been reported to have several possible beneficial effects, e.g., anti-carcinogenic, anti-ulcer, anti-thrombotic, anti-inflammatory, anti-allergenic, immunomodulating, antimicrobial, vasodilatory, analgesic, or antidiabetic effects [16,22].

The beverages studied in the present work were found to contain considerably large quantities of various polyphenols and thus could exert a particular functional effect on the human body through the different properties of their polyphenols. Amongst these features, antioxidant and antidiabetic properties were studied here to evaluate the potential bioactivity of the beverages.

#### 3.4.1. Antioxidant Capacity

The development of some chronic diseases such as cancer, cardiovascular diseases, and diabetes is tightly related to oxidative stress. Therefore, new chemopreventive approaches have been developed for preventing the damaging effects of free radicals and oxidants, mostly based on acquisition of radical scavengers and antioxidants from the diet. It has been already demonstrated that polyphenols from cocoa products (mostly flavanols) can interfere with these harmful processes and thus prevent the pathogenesis of the aforementioned diseases [31].

Results showing the radical scavenging or antioxidant capacity of the functional beverages expressed in mmol TE/L are presented in Figure 1d. The highest antioxidant capacity values were found in the beverages produced by the Moka and the Neapolitan methods at GD4 (7.29 and 6.58 mmol TE/L, respectively). These values were at least twice higher than those observed in the other beverages. A general trend was observed with a proportional increase in the antioxidant capacity with the decreasing CBS particle size for all the beverages produced by percolation techniques, whereas those obtained by the French press manifested no dependence on the GD, having the highest level of antioxidant capacity at the bigger CBS particle sizes, as reported about other parameters above. Antioxidant capacity showed significantly high correlations with the obtained values of TPC, TFC, and TTC (*r* = 0.9656, *r* = 0.9716, and *r* = 0.9649, respectively), even though lower correlations were seen between the antioxidant capacity and the single compounds detected by HPLC (ranging from *r* = 0.7713 for quercetin-3-O-rhamnoside to *r* = 0.9348 for procyanidin B2).

#### 3.4.2. Antidiabetic Capacity

**Figure 2:**
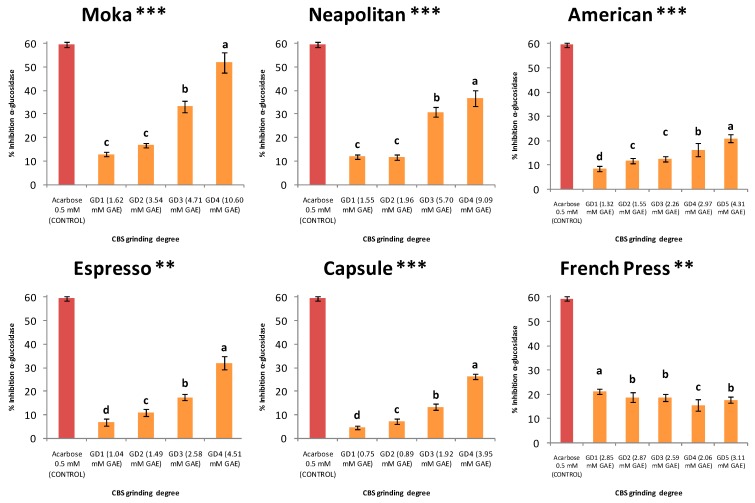
α-Glucosidase inhibition by the beverages produced via the six techniques at various CBS GDs related to the total phenolic content expressed in mM GAE (gallic acid equivalents); ANOVA among GDs for each technique. Acarbose at 0.5 mM (IC_50_) served as a control. Different letters indicate significant differences among the GDs at *p* < 0.05. Significance: ** *p* < 0.01; *** *p* < 0.001.

Cocoa polyphenols, in particular flavanols, have been reported to possess several antidiabetic bioactivities such as the improvement of insulin secretion by protecting β-pancreatic cells and the improvement of insulin sensitivity by protecting insulin-sensitive tissues from oxidative damage, among other reasons [9]. However, the main and more extended antidiabetic property of polyphenols have been reported to be the inhibition of key enzymes involved in glucose metabolism and absorption such as α-glucosidase or α-amylase [16]. This effect could be achieved by means of some drugs such as acarbose, a potent α-glucosidase inhibitor that is in disuse because of its considerable side effects that affect quality of life, e.g., abdominal distension, flatulence, meteorism, and possibly diarrhea [16]. For all these reasons, there is great interest among researchers in the search for healthy and sustainable alternatives such as the CBS preparations for beverages presented in this work. We carried out the study of the antidiabetic capacity due to the α-glucosidase inhibition of the beverages and the results are depicted in Figure 2.

As expected, according to TPC, TFC, and TTC results and taking into account the potential influence of these compounds on the antidiabetic capacity, the beverage exerting the higher percentage of α-glucosidase inhibition was the one obtained with Moka at the smallest CBS particle size GD4 (52.0%), followed by the Neapolitan (36.8%), Espresso (32.0%), and Capsule (26.2%) beverages at the same GD. The smallest percentage of α-glucosidase inhibition was observed for the beverage produced by the Capsule technique at the biggest GD of the CBS (4.7% with GD1). In general, beverages produced with big particle sizes of the CBS are those exerting the smallest α-glucosidase inhibition, especially for the techniques that employ pressure for extraction, where the contact time between water and the CBS decreases, and so does extraction performance. The French press beverages instead showed an intermediate value of α-glucosidase inhibition independently of the CBS particle size. It is important to note that the contribution to the antidiabetic effect manifested by the new functional beverages is close to that of 0.5 mM acarbose serving as a control sample corresponding to the IC_50_ concentration for this drug. The α-glucosidase inhibition parameters showed a significantly high correlation with TPC (*r* = 0.9537) and some of the detected polyphenolic compounds such as protocatechuic acid (*r* = 0.9826), type B procyanidin (*r* = 0.9870), and, as expected, both flavan-3-ols catechin-3-O-glucoside and epicatechin (*r* = 0.9803 and *r* = 0.9500, respectively).

This is the first study where the α-glucosidase inhibition due to polyphenolic content is reported for CBS. Nonetheless, the hypoglycemic effects of this by-product due to its fiber content have already been described by Nsor-Atindana et al. [32]. We did not evaluate this effect.

### 3.5. Consumer Acceptance Evaluation

Table 4 shows the sums of ranks values calculated for the consumer evaluation parameters for each beverage preparation technique and CBS particle size. These values were subjected to the Kruskal–Wallis test to highlight the differences in acceptance for the different beverages prepared by the same technique, comparing the GDs of the CBS.

As far as the aspect was concerned, slight differences were evidenced, and a general preference for the beverages obtained with smaller GDs was observed. Taking into account the results obtained for the beverages’ color, where in general, the brightness decreased while the GD decreased and both parameters a* and b* rose when the CBS GD decreased, it could be assumed that darker and browner beverages were preferred over lighter ones. Regarding the odor, the beverages obtained by the Neapolitan and the Moka techniques were generally the most appreciated while those obtained by the Capsule and the French press techniques were the least appreciated. In the case of taste, the beverage obtained by the Capsule technique showed high levels of consumer acceptance for almost all of the GDs. In some cases, even the beverages obtained by the Neapolitan and American methods were highly liked. The situation was again ambiguous for the flavor where the liking seems to be influenced by the technique–GD interaction, though in general, the beverages obtained by the Neapolitan or American technique were among the most appreciated. It was observed that overall, the beverages that had high values of titratable acidity were less liked in terms of taste and flavor. The overall rating obviously reflects all the previous variability for the various parameters of consumer acceptance, with the Neapolitan and the American techniques yielding the most liked beverages at the highest GDs, whereas the Capsule and Espresso methods seem to be the ones preferred at small GDs. Again, this pattern is reflected in the purchase predisposition, with a greater preference for Neapolitan technique beverages with large CBS particle sizes and for those generated by Capsule and Espresso at smaller GDs.

It should be remarked that the most active beverages (Moka and Neapolitan with small GDs) are between those most appreciated as far as the appearance or the odor are concerned. But, on the contrary, the appreciation of these beverages decreases considerably when evaluating taste and flavor, and so do the overall liking and the purchase predisposition. This fact could be related to the unpleasant, bitter and, in some cases, astringent flavor related to the polyphenols and the methylxantines which are present in high amounts in these beverages.

### 3.6. Technological Efficiency of Polyphenol Extraction

To identify the technique allowing higher extraction yield of polyphenols, normalization of the TPC values was performed according to the different CBS powder amounts and water quantities employed. The new TPC values, expressed in milligrams of gallic acid equivalents per gram of CBS ranged between 2.72 and 16.32 mg GAE/g (Table 5). A significant increase in TPC was observed for the beverages obtained by the forced percolation techniques (Moka, Espresso, and Capsule) at GDs below 1000 μm and for the beverages produced by the Neapolitan method (natural percolation) at GDs below 2000 μm. For the beverage produced by the American technique (natural percolation), TPC increased progressively with the decreasing GDs. On the contrary, for the beverage produced with the French press, no influence of the GDs was observed, thereby leading to the conclusion that the difference in GDs had only a minor impact on the polyphenol extraction by this technique based on a maceration process. Neapolitan was always the most effective technique when total phenol extraction was compared among the beverages obtained by all the techniques at the same GD, followed by those produced by the American, French press, or Moka techniques. The lowest TPC values belong to the beverages produced by the Capsule and Espresso. On the other hand, these techniques usually afforded higher extraction of essential oils because they produce more aromatic coffee. The highest TPC observed (16.32 mg GAE/g for the beverage obtained with Neapolitan using GD4) was significantly higher than those reported for other types of CBS extraction in previous works. Hernández-Hernández et al. [23] obtained a TPC value of 3 mg GAE/g when extracting 1 g of CBS at 500 μm in 6 mL of water at 70 °C. Manzano et al. [33] observed a TPC value of 6.04 mg GAE/g using 2 g of CBS screened at 75 μm, which was processed in 50 mL of water with 5-min reflux extraction. Nevertheless, in other studies in which assisted extraction was carried out, higher values of TPC were obtained as compared to those obtained for the beverages in the present work, as expected. Nsor-Atindana et al. [32] reported a value of 17.21 mg GAE/g after the extraction of 2 g of CBS ground up at 250 μm in 50 mL of water using microwaves. In any case, it is important to note that the solid/liquid ratio, which was different in all studies, could also have some influence on these results.

## 4. Conclusions

The various extraction techniques used for CBS ground to different degrees allowed us to obtain beverages with different chemical characteristics and consumer-related parameters. Several compounds were identified and quantified by HPLC (phenolic acids, flavan-3-ols, quercetin-glycosides, catechin-glycosides, and procyanidins), which may underlie the high radical scavenging capacity and significant α-glucosidase inhibition results shown by the beverages. The GD was optimized for each extraction technique; the smallest GDs allowed us to obtain the most functional beverages when using percolation techniques, whereas the maceration technique (French press) in general, showed no dependence on the CBS particle size. This finding may be of great interest as with the French press technique, no further CBS grinding treatments will be needed to obtain a beverage having high-potential biological activities. In terms of consumer acceptance, it was found that, in general, the most active beverages were the least appreciated as far as taste and flavor are concerned, probably because of the bigger presence of polyphenols and methylxanhines. This fact could open a possibility for further research in order to optimize these beverages, aiming at a higher consumer acceptance. Such optimization could be achieved by bioactive compounds encapsulation or by adding new pleasant ingredients, among other options.

For the first time, it was demonstrated that the Moka and Neapolitan techniques may be the most effective methods for polyphenol extraction, affording the highest radical scavenging activity and α-glucosidase inhibition capacity, whereas the beverage produced by the Capsule technique showed the poorest extraction. Therefore, this work indicates that CBS may be an optimal ingredient for home-made functional beverages with potential health benefits for consumers, thereby reducing the environmental and economic impact of by-product disposal.

## Figures and Tables

**Table 1:** Amounts of cocoa bean shell (CBS) powder and water utilized for the beverage preparations, volume of the obtained beverages, and process yield registered for each production technique and GD. Analysis of variance (ANOVA) was performed on the process yields among GDs and extraction techniques.

		Moka	Neapolitan	American	Espresso	Capsule	French press	*Sig.*
>4000 μm	Water (mL)	230.00	400.00	200	n/a	n/a	100.00	***
	CBS powder (g)	10.00	14.00	8.00	7.00	2.00	6.00	
	Beverage (mL)	188.00 ± 3.46	373.33 ± 2.89	172.00 ± 1.73	123.67 ± 1.53	59.33 ± 0.58	82.67 ± 0.58	
	Yield (%)	81.74 ± 1.51 ^bC^	93.33 ± 0.72 ^aA^	86.00 ± 0.87 ^aB^	n/a	n/a	82.67 ± 0.58 ^aC^	
2000–4000 μm	Water (mL)	230.00	400.00	200.00	n/a	n/a	100.00	***
	CBS powder (g)	18.00	17.00	8.00	13.00	2.80	6.00	
	Beverage (mL)	190.00 ± 2.00	363.33 ± 2.89	159.67 ± 6.81	125.67 ± 2.08	60.33 ± 0.58	83.00 ± 1.00	
	Yield (%)	82.61 ± >0.87 ^abBC^	90.83 ± 0.72 ^aA^	79.83 ± 3.40 ^bC^	n/a	n/a	83.00 ± 1.00 ^aB^	
1000–2000 μm	Water (mL)	230.00	400.00	200.00	n/a	n/a	100.00	***
	CBS powder (g)	22.00	30.00	8.00	16.00	6.50	6.00	
	Beverage (mL)	188.33 ± 1.53	329.66 ± 1.53	152.67 ± 1.15	122.67 ± 0.29	60.33 ± 0.58	78.67 ± 1.15	
	Yield (%)	81.88 ± 0.66 ^bA^	82.42 ± 0.38 ^aA^	76.33 ± 0.58 ^cC^	n/a	n/a	78.67 ± 1.15 ^bB^	
500–1000 μm	Water (mL)	230.00	400.00	200.00	n/a	n/a	100.00	***
	CBS powder (g)	26.00	30.00	8.00	18.00	8.00	6.00	
	Beverage (mL)	186.67 ± 2.31	316.67 ± 5.77	148.67 ± 4.16	119.67 ± 0.58	60.33 ± 0.58	72.00 ± 1.73	
	Yield (%)	81.16 ± 1.00 ^bA^	79.17 ± 1.44 ^aA^	74.33 ± 2.08 ^cB^	n/a	n/a	72.00 ± 1.73 ^cC^	
250–500 μm	Water (mL)	230.00	400.00	200.00	n/a	n/a	100.00	***
	CBS powder (g)	26.00	30.00	8.00	18.00	8.00	6.00	
	Beverage (mL)	68.00 ± 13.86	200.00 ± 81.85	146.67 ± 2.31	71.00 ± 9.64	36.35 ± 0.28	71.33 ± 1.53	
	Yield (%)	29.57 ± 6.02 ^cC^	50.00 ± 20.46 ^bB^	73.33 ± 1.15 ^cA^	n/a	n/a	71.33 ± 1.53 ^cA^	
*Sig.*		***	**	***	n/a	n/a	***	

n/a, not applicable. Means followed by different lower case superindexes within the same column (different grinding degrees) and by upper case superindexes within the same row (different techniques) are significantly different at *p* < 0.05. Significance: ** *p* < 0.01; *** *p* < 0.001. Data are expressed as mean values (*n* = 3) ± standard deviation.

**Table 2:** pH, titratable acidity, dry matter, and CIELab values of the beverages obtained by each extraction technique and grinding degrees (GD) of CBS, and ANOVA among GDs for each technique.

Technique	Grinding Degree (μm)	pH	Titratable acidity	Dry weight	L*	a*	b*
			(g acetic acid eq/L)	(%)			
Moka	>4000	4.91 ± 0.05 ^a^	0.40 ± 0.05 ^d^	0.52 ± 0.06 ^d^	82.73 ± 1.51 ^a^	5.69 ± 1.36 ^d^	50.84 ± 3.99 ^c^
	2000–4000	4.88 ± 0.01 ^a^	0.61 ± 0.05 ^c^	0.84 ± 0.11 ^c^	75.91 ± 0.64 ^b^	11.65 ± 1.06 ^c^	64.77 ± 2.83 ^b^
	1000–2000	4.89 ± 0.03 ^a^	0.95 ± 0.06 ^b^	1.53 ± 0.06 ^b^	61.20 ± 2.83 ^c^	25.76 ± 1.85 ^b^	80.92 ± 0.54 ^a^
	500–1000	4.93 ± 0.01 ^a^	1.64 ± 0.09 ^a^	2.70 ± 0.17 ^a^	33.21 ± 1.96 ^d^	37.75 ± 1.12 ^a^	55.08 ± 3.30 ^c^
	250–500	n/a	n/a	n/a	n/a	n/a	n/a
	*Significance*	ns	***	***	***	***	***
Neapolitan	>4000	4.92 ± 0.03 ^a^	0.29 ± 0.03 ^c^	0.41 ± 0.01 ^c^	82.76 ± 0.70 ^a^	5.52 ± 0.50 ^c^	48.74 ± 1.33 ^c^
	2000–4000	4.91 ± 0.04 ^a^	0.37 ± 0.04 ^c^	0.49 ± 0.06 ^c^	80.56 ± 2.80 ^a^	7.33 ± 2.19 ^c^	54.14 ± 5.51 ^c^
	1000–2000	4.88 ± 0.03 ^a^	0.95 ± 0.08 ^b^	1.50 ± 0.15 ^b^	56.56 ± 4.34 ^b^	28.45 ± 2.89 ^b^	79.31 ± 0.68 ^a^
	500–1000	4.93 ± 0.01 ^a^	1.23 ± 0.04 ^a^	2.00 ± 0.13 ^a^	39.04 ± 2.66 ^c^	37.24 ± 0.70 ^a^	63.97 ± 4.02 ^b^
	250–500	n/a	n/a	n/a	n/a	n/a	n/a
	*Significance*	ns	***	***	***	***	***
American	>4000	4.96 ± 0.01 ^b^	0.26 ± 0.01 ^d^	0.36 ± 0.01 ^c^	87.98 ± 0.53 ^a^	1.98 ± 0.18 ^e^	38.34 ± 0.68 ^e^
	2000–4000	5.07 ± 0.04 ^a^	0.37 ± 0.02 ^c^	0.51 ± 0.04 ^bc^	85.17 ± 0.64 ^b^	4.02 ± 0.51 ^d^	48.39 ± 1.86 ^d^
	1000–2000	5.07 ± 0.01 ^a^	0.40 ± 0.01 ^b^	0.69 ± 0.01 ^b^	80.12 ± 0.34 ^c^	8.35 ± 0.26 ^c^	59.79 ± 0.60 ^c^
	500–1000	4.99 ± 0.02 ^b^	0.55 ± 0.01 ^a^	1.01 ± 0.26 ^a^	73.99 ± 1.33 ^d^	14.54 ± 1.37 ^b^	71.05 ± 1.92 ^b^
	250–500	4.87 ± 0.01 ^c^	0.57 ± 0.01 ^a^	1.00 ± 0.04 ^a^	64.30 ± 0.74 ^e^	23.80 ± 0.72 ^a^	80.12 ± 0.31 ^a^
	*Significance*	***	***	***	***	***	***
Espresso	>4000	5.09 ± 0.07 ^a^	0.20 ± 0.02 ^d^	0.28 ± 0.04 ^d^	91.14 ± 1.37 ^a^	0.58 ± 0.69 ^d^	29.45 ± 3.67 ^d^
	2000–4000	4.98 ± 0.04 ^b^	0.37 ± 0.04 ^c^	0.51 ± 0.05 ^c^	86.15 ± 1.81 ^b^	3.32 ± 1.29 ^c^	44.39 ± 4.08 ^c^
	1000–2000	4.98 ± 0.01 ^b^	0.61 ± 0.05 ^b^	0.89 ± 0.07 ^b^	77.63 ± 2.16 ^c^	10.61 ± 2.13 ^b^	64.10 ± 3.74 ^b^
	500–1000	4.87 ± 0.02 ^c^	1.08 ± 0.02 ^a^	1.62 ± 0.05 ^a^	65.87 ± 0.52 ^d^	22.10 ± 0.51 ^a^	79.52 ± 0.69 ^a^
	250–500	n/a	n/a	n/a	n/a	n/a	n/a
	*Significance*	**	***	***	***	***	***
Capsule	>4000	5.19 ± 0.04 ^a^	0.12 ± 0.01 ^d^	0.15 ± 0.00 ^d^	94.73 ± 0.12 ^a^	−0.47 ± 0.05 ^c^	18.15 ± 0.56 ^d^
	2000–4000	5.15 ± 0.04 ^a^	0.17 ± 0.01 ^c^	0.24 ± 0.00 ^c^	92.73 ± 0.21 ^a^	−0.08 ± 0.06 ^c^	25.28 ± 0.90 ^c^
	1000–2000	5.15 ± 0.02 ^a^	0.43 ± 0.00 ^b^	0.66 ± 0.01 ^b^	82.41 ± 0.67 ^b^	6.22 ± 0.58 ^b^	53.72 ± 1.56 ^b^
	500–1000	4.84 ± 0.01 ^b^	0.93 ± 0.03 ^a^	1.33 ± 0.06 ^a^	68.71 ± 1.92 ^c^	19.06 ± 1.73 ^a^	75.91 ± 1.66 ^a^
	250–500	n/a	n/a	n/a	n/a	n/a	n/a
	*Significance*	***	***	***	***	***	***
French press	>4000	4.91 ± 0.05 ^ab^	0.62 ± 0.00 ^a^	0.91 ± 0.06 ^a^	77.73 ± 1.53 ^b^	10.80 ± 1.47 ^a^	67.09 ± 2.30 ^a^
	2000–4000	4.94 ± 0.02 ^ab^	0.65 ± 0.05 ^a^	0.91 ± 0.05 ^a^	76.48 ± 2.49 ^b^	11.97 ± 2.55 ^a^	68.31 ± 4.37 ^a^
	1000–2000	4.94 ± 0.01 ^ab^	0.50 ± 0.04 ^b^	0.86 ± 0.10 ^a^	77.42 ± 2.71 ^b^	10.96 ± 2.65 ^a^	65.83 ± 4.68 ^a^
	500–1000	4.96 ± 0.01 ^a^	0.40 ± 0.02 ^c^	0.66 ± 0.05 ^b^	82.23 ± 1.42 ^a^	6.53 ± 1.27 ^b^	56.03 ± 3.50 ^b^
	250–500	4.90 ± 0.02 ^b^	0.49 ± 0.03 ^b^	0.83 ± 0.05 ^a^	75.22 ± 0.36 ^b^	12.75 ± 1.40 ^a^	67.87 ± 3.18 ^a^
	*Significance*	ns	***	**	*	*	*

n/a, not applicable. Means followed by different letters are significantly different at *p* < 0.05. Significance: * *p* < 0.05; ** *p* < 0.01; *** *p* < 0.001; ns = not significant. Data are expressed as mean values (*n* = 3) ± standard deviation.

**Table 3:** Content (mg/L) of methylxanthines (theobromine and caffeine) and polyphenols (phenolic acids, flavan-3-ols, procyanidins B, and flavonols) evaluated by HPLC for the beverages obtained via the different techniques and GDs of the CBS and ANOVA among GDs for each technique.

Technique	Grinding Degree (μm)	Methylxanthines		Phenolic acids		Flavan-3-ol			Procyanidins B		Flavonols		
		Theobromine	Caffeine	Protocatechuic acid	Caffeic acid	Catechin-3-O-glucoside	Catechin	Epicatechin	Type B procyanidin	Procyanidin B2	Quercetin-3-O-glucoside	Quercetin-3-O-rhamnoside	Quercetin
Moka	>4000	147.33 ± 12.08 ^d^	25.44 ± 2.85 ^d^	5.25 ± 0.66 ^c^	0.17 ± 0.01 ^c^	6.43 ± 0.76 ^c^	1.01 ± 0.06 ^b^	0.51 ± 0.03 ^d^	8.64 ± 1.52 ^c^	0.79 ± 0.04 ^d^	0.19 ± 0.01 ^c^	0.24 ± 0.02 ^d^	0.88 ± 0.00 ^d^
	2000–4000	261.85 ± 12.05 ^c^	37.75 ± 2.38 ^c^	5.98 ± 0.95 ^c^	0.35 ± 0.05 ^b^	6.16 ± 0.19 ^c^	0.41 ± 0.02 ^b^	1.77 ± 0.02 ^c^	10.50 ± 1.80 ^c^	1.43 ± 0.17 ^c^	0.44 ± 0.02 ^b^	0.39 ± 0.07 ^c^	1.76 ± 0.01 ^c^
	1000–2000	384.78 ± 13.15 ^b^	68.07 ± 5.73 ^b^	10.29 ± 1.00 ^b^	0.41 ± 0.08 ^b^	13.49 ± 0.43 ^b^	0.81 ± 0.09 ^b^	3.20 ± 0.18 ^b^	20.38 ± 3.03 ^b^	1.81 ± 0.32 ^b^	0.50 ± 0.04 ^b^	0.51 ± 0.02 ^b^	1.80 ± 0.01 ^b^
	500–1000	703.79 ± 52.64 ^a^	124.84 ± 15.91 ^a^	18.14 ± 1.38 ^a^	0.81 ± 0.05 ^a^	21.73 ± 1.98 ^a^	1.92 ± 0.33 ^a^	6.38 ± 0.10 ^a^	36.30 ± 5.29 ^a^	3.30 ± 0.13 ^a^	0.93 ± 0.13 ^a^	0.66 ± 0.04 ^a^	3.54 ± 0.02 ^a^
	250–500	n/a	n/a	n/a	n/a	n/a	n/a	n/a	n/a	n/a	n/a	n/a	n/a
	*Sig.*	***	***	***	***	***	***	***	***	***	***	***	***
Neapolitan	>4000	127.84 ± 3.97 ^d^	19.17 ± 0.80 ^c^	3.44 ± 0.15 ^c^	0.16 ± 0.00 ^d^	4.21 ± 0.06 ^b^	0.59 ± 0.08 ^bc^	0.45 ± 0.01 ^d^	5.56 ± 0.06 ^c^	0.74 ± 0.03 ^d^	0.17 ± 0.01 ^d^	0.17 ± 0.01 ^d^	0.88 ± 0.01 ^d^
	2000–4000	185.78 ± 12.03 ^c^	26.11 ± 2.15 ^c^	4.10 ± 0.38 ^c^	0.31 ± 0.01 ^c^	4.61 ± 0.27 ^b^	0.36 ± 0.02 ^c^	1.55 ± 0.04 ^c^	7.29 ± 0.46 ^c^	1.30 ± 0.04 ^c^	0.36 ± 0.03 ^c^	0.32 ± 0.00 ^c^	1.75 ± 0.00 ^c^
	1000–2000	418.97 ± 24.11 ^b^	80.49 ± 8.83 ^b^	10.94 ± 1.55 ^b^	0.40 ± 0.04 ^b^	13.67 ± 0.67 ^a^	0.83 ± 0.20 ^b^	3.61 ± 0.04 ^b^	20.69 ± 2.15 ^b^	1.59 ± 0.10 ^b^	0.51 ± 0.04 ^b^	0.52 ± 0.04 ^b^	1.78 ± 0.00 ^b^
	500–1000	583.12 ± 29.74 ^a^	102.98 ± 7.30 ^a^	13.23 ± 0.88 ^a^	0.67 ± 0.01 ^a^	13.58 ± 1.13 ^a^	1.57 ± 0.20 ^b^	5.24 ± 0.16 ^a^	27.10 ± 1.90 ^a^	2.88 ± 0.08 ^a^	0.98 ± 0.07 ^a^	0.75 ± 0.01 ^a^	3.54 ± 0.02 ^a^
	250–500	n/a	n/a	n/a	n/a	n/a	n/a	n/a	n/a	n/a	n/a	n/a	n/a
	*Sig.*	***	***	***	***	***	***	***	***	***	***	***	***
American	>4000	115.00 ± 3.29 ^e^	14.57 ± 1.42 ^e^	2.91 ± 0.24 ^e^	0.15 ± 0.00 ^c^	3.89 ± 0.28 ^c^	0.36 ± 0.08 ^c^	0.25 ± 0.03 ^b^	5.71 ± 0.14 ^c^	0.72 ± 0.03 ^c^	0.16 ± 0.00 ^d^	0.15 ± 0.00 ^b^	0.87 ± 0.00 ^b^
	2000–4000	145.57 ± 5.42 ^d^	21.24 ± 1.26 ^d^	4.20 ± 0.23 ^d^	0.17 ± 0.01 ^c^	5.95 ± 0.23 ^b^	0.52 ± 0.04 ^bc^	0.43 ± 0.07 ^b^	7.84 ± 0.21 ^b^	0.77 ± 0.06 ^c^	0.19 ± 0.00 ^c^	0.15 ± 0.01 ^b^	0.87 ± 0.00 ^b^
	1000–2000	233.01 ± 3.92 ^a^	34.38 ± 0.97 ^c^	5.54 ± 0.07 ^c^	0.33 ± 0.03 ^a^	6.17 ± 0.26 ^b^	0.39 ± 0.03 ^bc^	1.30 ± 0.06 ^a^	10.54 ± 0.86 ^a^	1.37 ± 0.04 ^a^	0.37 ± 0.01 ^a^	0.35 ± 0.03 ^a^	1.75 ± 0.01 ^a^
	500–1000	180.89 ± 1.11 ^c^	37.09 ± 0.72 ^b^	6.76 ± 0.30 ^b^	0.22 ± 0.02 ^b^	8.65 ± 0.26 ^a^	1.08 ± 0.05 ^a^	1.54 ± 0.43 ^a^	12.19 ± 0.58 ^a^	0.99 ± 0.09 ^b^	0.20 ± 0.02 ^c^	0.17 ± 0.01 ^b^	0.88 ± 0.00 ^b^
	250–500	193.03 ± 0.84 ^b^	45.69 ± 0.91 ^a^	7.23 ± 0.27 ^a^	0.21 ± 0.01 ^b^	8.44 ± 0.56 ^a^	0.55 ± 0.16 ^b^	1.65 ± 0.13 ^a^	12.33 ± 2.33 ^a^	0.97 ± 0.06 ^b^	0.24 ± 0.02 ^b^	0.16 ± 0.01 ^b^	0.87 ± 0.00 ^b^
	*Sig.*	***	***	***	***	***	***	***	***	***	***	***	***
Espresso	>4000	92.34 ± 9.99 ^d^	10.63 ± 0.81 ^d^	2.10 ± 0.13 ^d^	0.16 ± 0.01 ^c^	2.67 ± 0.21 ^d^	0.24 ± 0.05 ^c^	0.04 ± 0.01 ^c^	4.16 ± 0.27 ^d^	0.65 ± 0.02 ^c^	0.17 ± 0.00 ^c^	0.17 ± 0.01 ^d^	0.87 ± 0.00 ^c^
	2000–4000	195.25 ± 20.65 ^c^	24.91 ± 3.64 ^c^	4.27 ± 0.54 ^c^	0.32 ± 0.01 ^b^	4.76 ± 0.54 ^c^	0.28 ± 0.03 ^c^	1.42 ± 0.10 ^b^	7.25 ± 1.23 ^c^	1.29 ± 0.02 ^b^	0.36 ± 0.02 ^b^	0.34 ± 0.02 ^c^	1.74 ± 0.00 ^b^
	1000–2000	292.82 ± 21.28 ^b^	42.90 ± 4.16 ^b^	7.16 ± 0.48 ^b^	0.32 ± 0.00 ^b^	9.43 ± 0.53 ^b^	0.53 ± 0.01 ^a^	1.84 ± 0.16 ^b^	13.97 ± 0.97 ^b^	1.34 ± 0.13 ^b^	0.38 ± 0.05 ^b^	0.45 ± 0.00 ^b^	1.76 ± 0.01 ^a^
	500–1000	392.36 ± 4.40 ^a^	66.81 ± 1.55 ^a^	12.59 ± 0.18 ^a^	0.43 ± 0.00 ^a^	13.98 ± 0.22 ^a^	0.45 ± 0.04 ^b^	2.87 ± 0.10 ^a^	20.72 ± 0.66 ^a^	1.60 ± 0.08 ^a^	0.45 ± 0.03 ^a^	0.56 ± 0.05 ^a^	1.77 ± 0.00 ^a^
	250–500	n/a	n/a	n/a	n/a	n/a	n/a	n/a	n/a	n/a	n/a	n/a	n/a
	*Sig.*	***	***	***	***	***	***	***	***	***	***	***	***
Capsule	>4000	59.82 ± 2.66 ^d^	6.09 ± 0.50 ^d^	1.32 ± 0.05 ^d^	0.14 ± 0.00 ^b^	1.71 ± 0.17 ^d^	0.12 ± 0.04 ^c^	0.00 ± 0.00 ^c^	2.77 ± 0.11 ^d^	0.60 ± 0.02 ^b^	0.15 ± 0.01 ^c^	0.17 ± 0.00 ^c^	0.86 ± 0.00 ^b^
	2000–4000	88.78 ± 3.27 ^c^	9.75 ± 0.59 ^c^	2.13 ± 0.11 ^c^	0.15 ± 0.00 ^b^	2.61 ± 0.03 ^c^	0.22 ± 0.03 ^bc^	0.14 ± 0.02 ^c^	3.96 ± 0.31 ^c^	0.68 ± 0.04 ^b^	0.15 ± 0.01 ^c^	0.13 ± 0.01 ^c^	0.87 ± 0.00 ^b^
	1000–2000	230.27 ± 9.07 ^b^	33.46 ± 0.15 ^b^	5.49 ± 0.36 ^b^	0.40 ± 0.07 ^a^	6.26 ± 0.33 ^b^	0.31 ± 0.09 ^ab^	1.27 ± 0.01 ^b^	9.49 ± 0.25 ^b^	1.31 ± 0.08 ^a^	0.35 ± 0.01 ^b^	0.39 ± 0.02 ^b^	1.76 ± 0.00 ^a^
	500–1000	361.79 ± 5.64 ^a^	59.60 ± 1.99 ^a^	10.80 ± 0.33 ^a^	0.38 ± 0.01 ^a^	12.21 ± 0.59 ^a^	0.37 ± 0.03 ^a^	2.53 ± 0.11 ^a^	17.99 ± 1.03 ^a^	1.39 ± 0.04 ^a^	0.44 ± 0.02 ^a^	0.49 ± 0.04 ^a^	1.76 ± 0.01 ^a^
	250–500	n/a	n/a	n/a	n/a	n/a	n/a	n/a	n/a	n/a	n/a	n/a	n/a
	*Sig.*	***	***	***	***	***	**	***	***	***	***	***	***
French press	>4000	289.14 ± 10.27 ^a^	43.07 ± 3.46 ^ab^	7.51 ± 0.28 ^a^	0.33 ± 0.01 ^b^	8.40 ± 0.57 ^a^	1.00 ± 0.03 ^a^	2.35 ± 0.23 ^a^	14.05 ± 0.79 ^a^	1.55 ± 0.05 ^a^	0.41 ± 0.02 ^a^	0.44 ± 0.04 ^a^	1.76 ± 0.01 ^a^
	2000–4000	272.30 ± 11.82 ^a^	40.47 ± 2.09 ^b^	7.34 ± 0.24 ^a^	0.34 ± 0.03 ^b^	8.16 ± 0.59 ^a^	0.54 ± 0.02 ^b^	1.77 ± 0.18 ^b^	13.58 ± 1.35 ^a^	1.61 ± 0.10 ^a^	0.41 ± 0.07 ^a^	0.39 ± 0.04 ^a^	1.76 ± 0.01 ^a^
	1000–2000	172.48 ± 9.12 ^b^	31.03 ± 4.31 ^c^	6.54 ± 0.63 ^b^	0.20 ± 0.00 ^c^	8.82 ± 1.09 ^a^	0.94 ± 0.08 ^a^	0.88 ± 0.11 ^c^	12.99 ± 1.82 ^a^	0.87 ± 0.02 ^b^	0.18 ± 0.02 ^b^	0.20 ± 0.03 ^b^	0.88 ± 0.00 ^b^
	500–1000	154.85 ± 22.81 ^b^	26.73 ± 2.38 ^c^	5.06 ± 0.22 ^c^	0.21 ± 0.01 ^c^	5.68 ± 0.19 ^b^	0.52 ± 0.05 ^b^	0.63 ± 0.03 ^c^	8.68 ± 0.74 ^b^	1.02 ± 0.21 ^b^	0.18 ± 0.01 ^b^	0.18 ± 0.02 ^b^	0.88 ± 0.01 ^b^
	250–500	286.68 ± 16.56 ^a^	46.65 ± 3.02 ^a^	7.70 ± 0.33 ^a^	0.36 ± 0.00 ^a^	7.80 ± 0.65 ^a^	0.57 ± 0.07 ^b^	2.27 ± 0.09 ^a^	12.59 ± 1.26 ^a^	1.44 ± 0.08 ^a^	0.42 ± 0.05 ^a^	0.39 ± 0.02 ^a^	1.76 ± 0.01 ^a^
	*Sig.*	***	***	***	***	**	*	*	**	***	***	***	***

n/a, not applicable. Means followed by different letters are significantly different at *p* < 0.05. Significance: * *p* < 0.05; ** *p* < 0.01; *** *p* < 0.001. Data are expressed as mean values (*n* = 3) ± standard deviation.

**Table 4:** Consumer evaluation of the beverages and results of the Kruskal–Wallis test. Data are expressed as the sum of ranks of the results obtained from 20 tasters who filled out a nine-point hedonic scale (1 = extremely dislike, 9 = extremely like).

Production Technique	Grinding Degree	Appearance	Odor	Taste	Flavor	Overall liking	Purchase predisposition
Moka	>4000 μm	309.5 ^ab^	234.0 ^b^	482.5 ^a^	473.0 ^a^	486.5 ^a^	395.0 ^a^
	2000–4000 μm	188.0 ^b^	220.0 ^b^	335.0 ^ab^	283.5 ^b^	312.5 ^b^	265.5 ^a^
	1000–2000 μm	288.5 ^ab^	311.5 ^ab^	206.0 ^bc^	188.0 ^b^	177.5 ^b^	279.0 ^a^
	500–1000 μm	390.0 ^a^	410.5 ^a^	152.5 ^c^	231.5 ^b^	199.5 ^b^	236.5 ^a^
	250–500 μm	n/a	n/a	n/a	n/a	n/a	n/a
	*Significance*	*	*	***	***	***	ns
Neapolitan	>4000 μm	372.5 ^a^	291.0 ^a^	449.5 ^a^	362.0 ^ab^	433.5 ^a^	439.5 ^a^
	2000–4000 μm	230.5 ^a^	344.5 ^a^	361.0 ^ab^	382.5 ^a^	406.0 ^a^	376.5 ^a^
	1000–2000 μm	271.0 ^a^	231.5 ^a^	245.5 ^bc^	202.5 ^b^	181.5 ^b^	205.0 ^b^
	500–1000 μm	302.0 ^a^	309.0 ^a^	120.0 ^c^	229.0 ^ab^	155.0 ^b^	155.0 ^b^
	250–500 μm	n/a	n/a	n/a	n/a	n/a	n/a
	*Significance*	ns	ns	***	**	***	***
American	>4000 μm	138.5 ^b^	338.0 ^a^	400.5 ^ab^	446.0 ^a^	436.0 ^ab^	467.5 ^a^
	2000–4000 μm	510.5 ^a^	444.0 ^a^	595.0 ^a^	557.5 ^a^	569.5 ^a^	403.0 ^a^
	1000–2000 μm	380.5 ^a^	288.0 ^a^	328.0 ^bc^	342.0 ^a^	309.0 ^bc^	318.0 ^a^
	500–1000 μm	461.0 ^a^	310.0 ^a^	363.0 ^bc^	396.0 ^a^	392.0 ^ab^	372.0 ^a^
	250–500 μm	339.5 ^ab^	450.0 ^a^	143.5 ^c^	88.5 ^b^	123.5 ^c^	269.5 ^a^
	*Significance*	***	ns	***	***	***	ns
Espresso	>4000 μm	120.0 ^b^	138.0 ^b^	277.0 ^ab^	247.0 ^ab^	172.5 ^b^	214.0 ^bc^
	2000–4000 μm	316.0 ^a^	337.0 ^a^	413.0 ^a^	369.0 ^a^	402.0 ^a^	394.5 ^a^
	1000–2000 μm	356.5 ^a^	290.0 ^ab^	366.5 ^a^	391.5 ^a^	351.5 ^a^	368.5 ^ab^
	500–1000 μm	383.5 ^a^	410.0 ^a^	119.5 ^b^	168.0 ^b^	250.0 ^ab^	199.0 ^c^
	250–500 μm	n/a	n/a	n/a	n/a	n/a	n/a
	*Significance*	***	***	***	**	**	**
Capsule	>4000 μm	125.5 ^b^	151.0 ^b^	431.0 ^b^	292.5 ^ab^	235.5 ^b^	201.0 ^b^
	2000–4000 μm	185.0 ^b^	248.0 ^b^	321.0 ^b^	188.0 ^b^	195.5 ^b^	206.0 ^b^
	1000–2000 μm	389.0 ^a^	310.0 ^ab^	172.0 ^ab^	283.5 ^ab^	300.0 ^ab^	339.5 ^ab^
	500–1000 μm	476.0 ^a^	467.0 ^a^	252.0 ^a^	412.0 ^a^	445.0 ^a^	429.5 ^a^
	250–500 μm	n/a	n/a	n/a	n/a	n/a	n/a
	*Significance*	***	***	***	**	***	***
French press	>4000 μm	464.5 ^a^	526.5 ^a^	192.0 ^c^	472.0 ^a^	436.0 ^a^	319.0 ^ab^
	2000–4000 μm	312.0 ^a^	172.0 ^b^	237.5 ^bc^	119.0 ^b^	132.5 ^b^	224.5 ^b^
	1000–2000 μm	366.0 ^a^	140.5 ^b^	367.5 ^abc^	290.0 ^ab^	285.5 ^ab^	493.0 ^a^
	500–1000 μm	265.5 ^a^	494.0 ^a^	580.0 ^a^	503.0 ^a^	488.0 ^a^	461.5 ^a^
	250–500 μm	422.0 ^a^	497.0 ^a^	453.0 ^ab^	446.0 ^a^	488.0 ^a^	332.0 ^ab^
	*Significance*	ns	***	***	***	***	**

n/a, not applicable. Means followed by different letters are significantly different at *p* < 0.05. Significance: * *p* < 0.05; ** *p* < 0.01; *** *p* < 0.001; ns = not significant. Data are expressed as sums of ranks.

**Table 5:** Total phenolic content for the beverages after normalization considering CBS and water quantities. ANOVA among GDs and extraction techniques. Values are expressed in milligrams of gallic acid equivalents for each gram of CBS powder employed for the beverage preparation (mg GAE/g CBS).

	Moka	Neapolitan	American	Espresso	Capsule	French Press	*Sig.*
>4000 μm	5.21 ± 0.54 ^cB^	7.03 ± 0.47 ^cA^	4.84 ± 0.28 ^dB^	3.12 ± 0.30 ^bC^	3.76 ± 0.25 ^bC^	6.68 ± 0.64 ^aA^	***
2000–4000 μm	6.37 ± 2.25 ^bcAB^	7.14 ± 1.06 ^cA^	5.26 ± 0.09 ^dB^	2.72 ± 0.39 ^bC^	3.25 ± 0.30 ^cC^	6.74 ± 0.72 ^aA^	***
1000–2000 μm	6.92 ± 0.72 ^bB^	10.00 ± 1.33 ^bA^	7.32 ± 0.34 ^cB^	3.41 ± 0.33 ^bD^	3.03 ± 0.11 ^cD^	5.78 ± 0.30 ^bC^	***
500–1000 μm	12.94 ± 0.96 ^aB^	16.32 ± 1.04 ^aA^	9.39 ± 0.92 ^bC^	5.10 ± 0.15 ^aD^	5.06 ± 0.25 ^aD^	4.10 ± 0.31 ^cE^	***
250–500 μm	n/a	n/a	13.45 ± 0.86 ^aA^	n/a	n/a	6.29 ± 0.26 ^aB^	***
*Sig.*	***	***	***	***	***	***	***

n/a, not applicable. Means followed by different lower case superindexes within the same column (different grinding degrees) and by upper case superindexes within the same row (different techniques) are significantly different at *p* < 0.05. Significance: *** *p* < 0.001. Data are expressed as mean values (*n* = 3) ± standard deviation.

## Abbreviations

CBS, cocoa bean shell; GD, grinding degree; TPC, total phenolic content; TFC, total flavonoid content; TTC, total tannin content, RSA, radical scavenging activity; GAE, gallic acid equivalents; CE, catechin equivalents; TE, trolox equivalents; RP-HLPC-PDA, reversed-phase high-pressure liquid chromatography equipped with photodiode array detector; PB1, procyanidin B1; PB2, procyanidin B2; Q-3-G, quercetin-3-O-glucoside; ANOVA, analysis of variance.
